# Neuropsychiatric manifestations of monkeypox: A clinically oriented comprehensive review

**DOI:** 10.1002/brb3.2934

**Published:** 2023-02-27

**Authors:** Yashendra Sethi, Pratik Agarwal, Hamsa Murli, Summaiya Waheed, Sajeda Ghassan Matar, Mohamed Baklola, Hitesh Chopra, Talha Bin Emran, Elfatih A. Hasabo

**Affiliations:** ^1^ Department of Medicine Government Doon Medical College Dehradun India; ^2^ Department of Medicine Lokmanya Tilak Municipal Medical College Mumbai India; ^3^ Department of Medicine Dow Medical College, Dow University Of Health Sciences Karachi Pakistan; ^4^ Faculty of Pharmacy Applied Science Private University Amman Jordan; ^5^ Faculty of medicine Mansoura University Mansoura Egypt; ^6^ Chitkara College of Pharmacy Chitkara University Punjab India; ^7^ Department of Pharmacy BGC Trust University Bangladesh Chittagong Bangladesh; ^8^ Department of Pharmacy, Faculty of Allied Health Sciences Daffodil International University Dhaka Bangladesh; ^9^ Faculty of Medicine University of Khartoum Khartoum Sudan

**Keywords:** monkeypox, MPX, MPX vaccine, neurological manifestations, outbreak, psychiatric manifestations

## Abstract

Monkeypox (MPX) has emerged as a threatening outbreak in recent months. The understanding of disease pathogenesis and its systemic involvement has evolved with time. Both the virus and its vaccine, like other members of the *Orthopoxvirus* family, were always expected to have neuropsychiatric consequences. Several neurological complications have been reported with MPX and its vaccines that include but not limited to headaches, myalgia, encephalitis, and coma. Psychiatric complications like anxiety and depression have also been reported; however, we lack evidence to present a direct causality. We conducted a literature review to compile recent evidence on neuropsychiatric manifestations and underline the importance of evolving aspects and complications of MPX. We advocate for better reporting of cases and adverse events, to enhance our understanding of the disease, aiding physicians to make more informed decisions, thus facilitating timely diagnosis and treatment.

## Graphical abstract

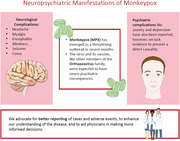

## 1 INTRODUCTION

The last 3 years have seen the emergence and re‐emergence of threatening viral diseases. The viral zoonosis of monkeypox (MPX), caused by an *Orthopoxvirus*, has presented a ghastly addition to the list. MPX has infected 72,457 cases in 109 countries globally with the milder west African clade of the virus dominating the outbreak (CDC, 2022a). It has been observed that the west African phylogenetic clade of MPX has a lower case fatality rate (3.6%) than the Congo Basin clade (10.6%) (WHO, 2022c). The World health organization (WHO) has declared the endemic disease of the African tropical rainforest a public health emergency of international concern (Farahat et al., 2022; WHO, 2022f).

MPV was first discovered in 1958 and was isolated from monkeys shipped to Denmark from Singapore (Cho & Wenner, 1973). However, the first case of zoonotic transmission to humans was reported in 1970 in the Democratic Republic of Congo (DRC) (Breman et al., 1980). In 2003, the first outbreak of MPX outside Africa was reported from the United States and was attributed to infected pet prairie dogs that were kept in contact with Gambian pouched rats from Ghana (WHO, 2022b). The communicable disease can spread and present in disparate ways. MPV can be transmitted through direct or indirect contact with infected skin lesions, body fluids, or respiratory droplets of animals or humans. Furthermore, transplacental transmission and sexual transmission have also been reported (Grant et al., 2020). During the 2022 outbreak, the first reported case of MPX in nonendemic regions was a traveler returning to the United Kingdom from Nigeria on 7th May. Other cases were reported independently during a similar time frame. This was followed by a rapid uptick in cases, suggesting human‐to‐human transmission. Epidemiological data suggested a substantially higher risk in men, particularly gay, bisexual, and other men who have sex with men (GBMSM) (Global.health, 2022; GOV.UK, 2022; WHO, 2022d, 2022e). The incubation period mostly ranges from 7 to 14 days with the lesions starting from the oropharynx and then spreading throughout the body (Moore et al., 2022). Symptoms include the characteristic lymphadenopathy and mild smallpox‐like disease with fever, muscle aches, headache, lethargy, and back pain. The infection can broadly be divided into two phases: (1) the initial invasion phase (0−5 days marked by symptoms of fever, headache, lymphadenopathy, backache, myalgia, and asthenia) and (2) the eruptive phase marked by the classical rash. The rash is mainly concentrated on the face and extremities often extending to oral mucous membranes, genitalia, conjunctivae, and the cornea. It evolves sequentially from macules to papules, vesicles, pustules, and crusts that dry up and fall off. The symptoms can be potent in immunocompromised populations (WHO, 2022a). In cases of sexual transmission, anogenital lesions are predominant with sometimes no systemic symptoms. The rash starts as local pseudo‐pustules that develop into generalized heterogeneous lesions (Català et al., 2022). Traditional cases of MPX prior to the 2022 outbreak presented with a febrile prodrome prior to the onset of the rash, whereas more recent cases have shown a complete absence of the febrile symptoms or fever following the appearance of the rash (WHO, 2022d; Thornhill et al., 2022). A study conducted in 2017 in the Democratic Republic of Congo showed that hunters, farmers, and people living in lower quality housing were more susceptible to the virus, indicating that direct, prolonged contact with afflicted animals may be the main mode of transmission (Quiner et al., 2017). More recent evidence suggests that sexual transmission through local inoculation is the main mode of transmission in nonendemic areas, highlighting a shift from animal‐to‐human to human‐to‐human transmission (Bunge et al., 2022). A gathering in the Canary Islands of around 80,000 attendants for the Maspalomas gay pride festival was suggested to be the probable cause of increased transmission of MPX in the area due to high contact rates (Haider et al., 2022; Zumla et al., 2022). Several clinics in the United Kingdom reported that more than 90% of presenting cases affected people who identified as GBMSM, with a predominance of anogenital lesions. Around a quarter of the patients had a concomitant STI or were suffering from HIV (Girometti et al., 2022; Patel et al., 2022).

Virologically, the MPX virus is an enveloped double‐stranded DNA virus of the genus *Orthopoxvirus* belonging to the Poxviridae family (WHO, 2022b). The viral diseases owing to their systemic inflammatory process, neurotropism, and potential to cause direct viral injury often produce neuropsychiatric manifestations. To cite a few, the Chikungunya, Ebola, Hendra, Influenza, Marburg, and coronavirus have all presented with neurological complications (McEntire et al., 2021). Being an *Orthopoxvirus*, parallelism with the smallpox virus is expected from MPV. The common central nervous system (CNS) complications implicated with smallpox disease or vaccination include encephalomyelitis (de Vries, 1960), Guillain–Barré syndrome (GBS) (Kisch, 1958), acute cranial neuropathies (MILLER, 1953), poliomyelitis‐like syndrome, bell's palsy, and transverse myelitis (Sejvar, 2005). In the United States, 2.5 per million people have experienced neurologic problems, with postvaccinal encephalomyelitis (PVEM) being the most prevalent. PVEM presents as stupor, coma, seizure, and paraparesis, especially in older children and adults. Around 16% of cases have reported long‐term neurologic effects, while mortality rates amount to 1.5 per million per vaccination recipient (Abrahams & Kaufman, 2004). Similar neuropsychiatric complications have been reported for MPX as well (Jezek et al., 1987; Sejvar et al., 2004). The most common is “encephalitis” presenting with the expected constitutional symptoms (Shafaati & Zandi, 2022). It is unknown whether this encephalitis is caused by a direct spread or an autoimmune mechanism (Pastula et al., 2022).

In absence of a specific MPX vaccine, the use of smallpox vaccination for limiting the spread of MPX has been widely advocated but the known neurological complications of smallpox vaccines cannot be ignored, especially with the limited data. However, recent studies support that the addition of vaccines carries a very low risk of producing or exacerbating neurologic complications (Abrahams & Kaufman, 2004).

The neuropsychiatric manifestations of viral diseases have always been a feared yet ignored aspect. The literature is still evolving on MPX and its various systemic manifestations, but we must learn from our previous experiences and be better prepared for future outbreaks. The current review is an attempt to comprehensively compile the evidence on manifestations and suggest preventive and management strategies to help create awareness and reduce morbidity.

## 2 NEUROLOGICAL SYMPTOMS IN MPX PATIENTS

MPX like other viral illnesses has also presented with some neurological manifestations (Table 1), with the most common symptom being headache. These are seen in more than half of the MPX‐infected patients. Sepsis, blindness (Shafaati & Zandi, 2022), seizures, photophobia (Sepehrinezhad et al., 2022), and encephalitis have also been reported. Though uncommon, encephalitis and seizures are significant neurological complications of MPX, with a prevalence of about 3% (Badenoch et al., 2022). Some of the recently reported cases have even presented with MPX‐associated serious neurological symptoms. Two cases of MPX encephalitis were reported in young girls necessitating intubation and mechanical ventilation. The first patient, a 3‐year‐old girl, died on the second day of her hospitalization, and no cerebrospinal fluid (CSF) confirmation of MPX disease was possible. While the other girl, a 6‐year‐old who recovered after 14 days in the intensive care unit, had *Orthopoxvirus*‐reactive IgM in her CSF (Sepehrinezhad et al., 2022).

**TABLE 1:** Compilation of reports of neuro‐psychiatric manifestations of MPX

Serial no.	First author	Year of publication	Country	Study design	Sample size	Neurological manifestations	Psychiatric manifestation	Management	Outcome	Reference
1	Dimie Ogoina	2020	Nigeria	Retrospective review of case records	40	Headache (19) Myalgia (25) Seizure (1) Encephalitis (3) Photophobia (9)	Anxiety (11) Depression (11) Suicide (1)	Symptomatic management and supportive care according to institutional guidelines.	Deaths (5) Survival (35)	Ogoina et al., 2020
2	Dimie Ogoina	2019	Nigeria	Cross‐sectional study	18	Headache (12) Myalgia (5) Pain (5) Photophobia (3)	Suicide (1)	According to institutional guidelines.	Deaths (1) Survival (17)	Ogoina et al., 2019
3	Adesola Yinka‐Ogunleye	2019	Nigeria	Review of the epidemiological report	118	Headache (61) Myalgia (42) Photophobia (27)	NA	NA	Deaths (7) Survival (111)	Yinka‐Ogunleye et al., 2019
4	S. Akar	2020	Nigeria	Retrospective review of case records	165	Headache (78)	NA	NA	Deaths (9) Survival (156)	Akar et al., 2020
5	E. E. Eseigbe	2021	Nigeria	Case study	2	Headache (2)	NA	Both cases were admitted and treated with antibiotics, antihistamines, nonsteroidal anti‐inflammatory drugs, and multivitamins.	Admitted and then discharged after treatment.	Eseigbe et al., 2021
6	Christine M. Hughes	2021	Democratic Republic of Congo (DRC).	Surveillance study	134	Headache (99) Myalgia (90) Fatigue (115)	NA	NA	NA	Hughes et al., 2021
7	Phillip R. Pittman	2022	Democratic Republic of Congo (DRC).	Prospective observational study	216	Headache (49) Myalgia (15) Dizziness (3) Visual deficit (5) Confusion (4) Fatigue (11)	NA	NA	Deaths (3) Fetal deaths among admitted pregnant patients (four out of five patients) Survival (213)	Pittman et al., 2022
8	Z. Ježek	1987	Zaire/DRC	Surveillance study	282	Encephalitis (1) Coma (1)	NA	NA	Deaths (27) Survival (32)	Jezek et al., 1987
9	Boumandouki P	2007	Democratic Republic of Congo (DRC).	Surveillance study	81(8 confirmed)	Myalgia (2)	NA	NA	No deaths	Boumandouki et al., 2007
10	E. Kalthan	2016	Central African Republic	Descriptive study	12	Headache (2)	NA	NA	Fatality (25%), about 67% in children less than 10 years of age	Kalthan et al., 2016
11	Gregory D. Huhn	2005	United States	Surveillance study	34	Headache (23) Myalgia (19) Seizure (1) Confusion (2) Encephalitis (1)	NA	Nine patients were hospitalized and treated as inpatients, two of them were a 6‐year‐old girl and a 10‐year‐old girl admitted to intensive care unit.	No deaths	Huhn et al., 2005
12	Donita R. Croft	2007	United States	Outbreak investigation and cohort study	19	Headache (13)	NA	NA	No deaths	Croft et al., 2007
13	Kurt D. Reed	2004	United States	Outbreak investigation	11	Headache (11) Myalgia (1)	NA	Four patients were hospitalized. Nine patients received antibiotics (six received ciprofloxacin, and eight were given doxycycline). One patient received intravenous acyclovir, and two patients received valacyclovir. No patients received vaccinia immune globulin.	The disease was self‐limiting in all cases	Reed et al., 2004
14	Mary G. Reynolds	2006	United States	Review of outbreak	37	Headache (32) Myalgia (36)	NA	NA	NA	Reynolds et al., 2006
15	Michael G. Anderson	2003	United States	Case report	1	Headache (1) Myalgia (1) Fatigue (1)	NA	Intravenous diphenhydramine, lorazepam, and morphine for oropharyngeal pain. Intravenous ampicillin/sulbactam, 200 mg/kg/day divided every 6 h for the retropharyngeal phlegmon. Bacitracin cream was applied on the facial lesions to minimize scarring.	Discharged after a week of admission.	Anderson et al., 2003
16	James J. Sejvar	2004	United States	Case series	3	Headache (2) Seizure (1) Altered mental status (1) Delirium/encephalopathy (1) Encephalitis (1)	NA	One patient required lorazepam, intubation, and intensive care. She was given empiric intravenous ceftriaxone, acyclovir, phenobarbital, and midazolam.	Two patients did not require inpatient care and their disease remain self‐curing. The third patient, a 6‐year‐old child was discharged after a 16‐day stay in the intensive care unit where she was intubated.	Sejvar et al., 2004
17	Hugh Adler	2022	United Kingdom	Retrospective observational study	7	Headache (1) Pain (1)	Low mood (3) Emotional lability (1)	Two patients did not require any treatment. A patient with low mood and emotional lability was given a psychological consult Treatment of others varied between symptomatic management and antibiotic administration.	All seven of them recovered and survived.	Adler et al., 2022
18	Lynne A. Learned	2005	Republic of Congo (ROC)	Case series	11	Headache (1) Fatigue (2)	Irritability (2) Distress (4)	All but three of them required hospitalization. Treatment varied between symptomatic management and hospitalization.	All 11 of them recovered.	Bolanda et al., 2005
19	Mary G. Reynolds	2013	Republic of Congo (ROC)	Surveillance study	2	Headache (1) Fatigue (1)	NA	NA	NA	Reynolds et al., 2013

Encephalitis in MPX presents with pharyngitis, fever, headache, adenopathy, and a vesiculopapular rash that spreads rapidly throughout the body. MRI depicts diffuse edema, meningeal amplification, and signal hyperintensity in the thalamus and parietal cortex. This often presents with a slow wave activity on electroencephalogram. The findings indicate the probability of mixed cytotoxic and vasogenic brain edema (Sepehrinezhad et al., 2022). CSF examination in the reported cases has revealed polymorphonuclear dominant pleocytosis with typical glucose and protein levels (Shafaati & Zandi, 2022). Most of the reported cases of encephalitis have been attributed to the West African strain. However, it is imperative to understand that there is a huge gap in reporting of cases and testing of the responsible viral clade, thus conclusions still need further evaluation.

## 3 NEUROTROPISM AND NEUROINVASIVE NATURE OF THE MPX VIRUS

The evidence on the neurotropic effects of MPX is still growing, but our understanding of other Pox viruses and the recently reported literature on MPX support the idea (Chowdhury et al., 2022). MPX being detected in brain tissues of animals suggests a neuroinvasive propensity of the virus with its potential to cross the blood–brain barrier (Sepehrinezhad et al., 2022). The virus was found in the brain tissue of four rodent suspects in the form of MPXV DNA during the 2003 outbreak (Kulesh et al., 2004).

Reports from various parts of the world have indicated the neuroinvasive property of MPX. Cases from the midwestern United States have shown symptoms of headache, myalgia, and encephalitis with these patients developing MPX due to Prairie dog exposure (Sejvar et al., 2004). In Spain, two fatal neuroinvasive instances involving MPXV encephalitis were recently recorded. Two cases of MPXV with encephalitis appearing within 5–9 days were reported in the United States that hinted at either neuroinvasion or a parainfectious autoimmune mechanism. Both the reported findings are indicative of ADEM(Pastula et al., 2022).

**FIGURE 1:**
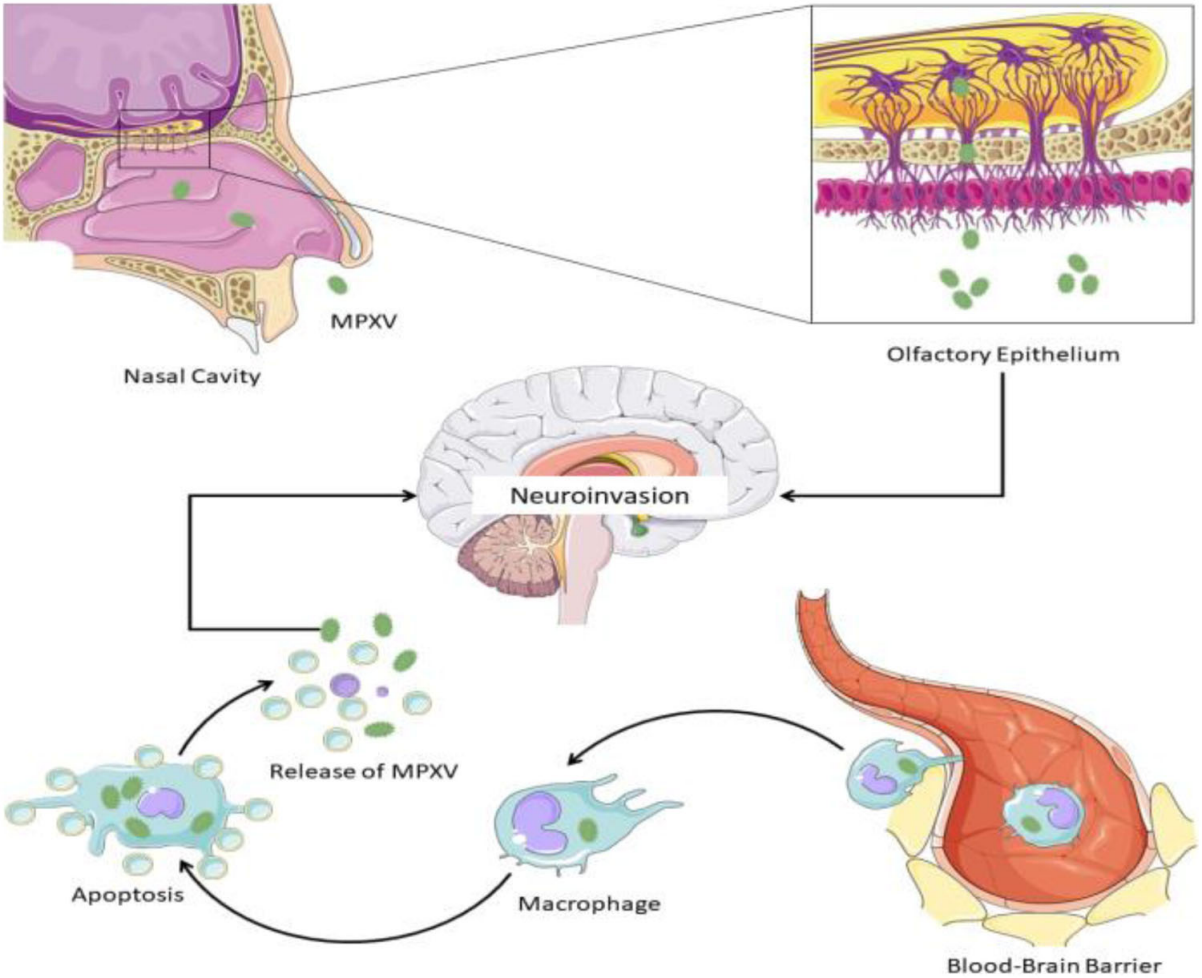
Probable neuroinvasive mechanism(s) of MPX. Parts of the figure were drawn using pictures from Servier Medical Art (smart.servier.com), provided by Servier, licensed under a Creative Commons Attribution 3.0 unported license (https://creativecommons.org/licenses/by/3.0/).

Furthermore, animal investigations have revealed two pathways for MPX transport to the CNS. One of these is through the olfactory epithelium. This was thought to be related to the remarkable build‐up of MPXV in the nasal septum and brain tissue following intranasal inoculation of Congo Basin MPXV stain in ground squirrels (Sergeev et al., 2017). Bioluminescence imaging also demonstrated fast viral replication in intranasal and cerebral tissues (Earl et al., 2015). Even in mice, the brain, nasal septum, and nasal mucosa have been shown to have a high viral load. The second route of infection to the CNS is thought to be blood‐borne transmission through infected monocytes/macrophages (Figure 1). This hypothesis is substantiated by the discovery of distinct MPXV‐Zaire 79 antigens in circulating monocytes after intravenous injection of the virus in macaques (Song et al., 2013). There are also reports that support an increased replication of these viruses in mediastinal and alveolar lymph nodes (Zaucha et al., 2001). Luciferase imaging to detect neurotropism in the virus could be useful in future research (Cook & Griffin, 2003).

## 4 PSYCHIATRIC SYMPTOMS IN MPX PATIENTS

MPX has been associated with some psychiatric manifestations as well. Anxiety and depression have been reported as the most commonly associated psychiatric manifestations. In Nigeria, around 25% of patients hospitalized for MPX were diagnosed with anxiety and depression (Ogoina et al., 2019). Three out of seven admitted patients with MPX in the United Kingdom had a low mood, as reported in a case series (Adler et al., 2022). In the Nigerian study, 11 patients out of 40 were reported to have anxiety and depression and required psychological counseling (Ogoina et al., 2019). One patient died by suicide due to the fear of the stigma associated with the illness and the route of transmission, though prior psychiatric history could not be established (Ogoina et al., 2019).

A catena of causes could be responsible for the said picture including but not limited to patient isolation, pain, skin lesions, and stigma. In both studies reporting psychiatric complications, the patients were isolated for infection control, which could have contributed to the low mood. Pain could also be one of the potential contributors. In the study from the United Kingdom, one out of three patients with low mood had severe pain along with deep tissue abscesses, and another had an ulcerated inguinal lesion (Adler et al., 2022). The MPX rash is known to be initially painful but later becomes itchy as it crusts (CDC, 2022). In one study with participants who were predominantly men who had sex with men, 36% of patients had rectal pain and around 10% required hospitalization for pain management or penile edema (Patel et al., 2022). Pain and depression are closely intertwined, sharing a biochemical basis involving serotonergic and noradrenergic pathways, as evidenced by the pain‐relieving properties of serotonergic and norepinephrine antidepressants (IsHak et al., 2018).

Furthermore, MPX has been seen to be associated with rashes over large areas of the body, including the face, trunk, limbs, and genitals. Four patients in the Nigerian study feared potential permanent facial scarring due to the rashes (Ogoina et al., 2019). Facial scarring has been linked to long‐term affective disorders such as depression, especially if the causative incident was traumatic in nature (Rogers et al., 2020). Fear of discrimination and stigmatization among patients and healthcare workers was a prominent feature. In the Nigerian study, three patients initially refused to be admitted to the isolation ward, fearing stigmatization in their community (Ogoina et al., 2019).

In a U.K. study, a patient's landlord tried to evict them during their hospital stay, causing significant distress (Adler et al., 2022). These manifestations cannot be directly attributed to the disease process, but are rather attributable to factors associated with the social milieu. These findings are similar to those seen with severe coronavirus infections with cases reporting PTSD, depression, and anxiety in the post‐illness stage (Rogers et al., 2020). Consequentially, the psychiatric impacts of the disease are not just restricted to the patients but also to their families and society (Sethi et al., 2022).

Instead of being a specific consequence of MPX, psychiatric complications might be the result of the events surrounding such a diagnosis, including isolation, stigmatization, physical pain, and disfigurement. Nevertheless, they should be kept in mind while treating a patient with MPX.

## 5 DIFFERENTIAL DIAGNOSIS OF THE NEUROLOGIC AND PSYCHIATRIC MANIFESTATIONS OF MPX

Smallpox, belonging to the same genus of *Orthopoxvirus*, shares most of its clinical features and complications with MPX. Smallpox can present with fever, lethargy, headache, and delirium, even before the onset of rashes. Encephalopathy is common and acute perivenular demyelination was observed among those who died from smallpox (Cleri et al., 2003; Pickfordmarsden, 1934). It can present with delirium, hallucinations, and severe headaches. Eye complications leading to blindness have also been reported (Cleri et al., 2003). Interestingly, the smallpox vaccine has also been shown to cause certain neurological complications like headaches, seizures, cranial nerve palsy, GBS, hemiplegia, and coma. Encephalitis is a rare complication with a mortality of about 25% (McEntire et al., 2021; Michael Lane et al., 1970). Although most similar to MPX, other differentials are more likely owing to the eradication of smallpox in modern times. The last known case occurred in 1978 due to a laboratory accident (Geddes, 2006).

Another infectious disease with overlapping features of MPX is Varicella Zoster. Fever, postinfectious encephalitis, meningitis, convulsions, and stroke are some of the neurological manifestations of varicella (Table 2). Key differentiating features include post‐herpetic neuralgia and the less common acute cerebellar ataxia (Bozzola et al., 2012). The oral and genital lesions observed in MPX are similar to herpes simplex virus infections. Oral lesions are a prominent feature of herpes simplex virus type 1 (HSV‐1) infection (Arduino & Porter, 2007). Genital lesions can be caused by both HSV‐1 and HSV‐2, though HSV‐2 infection is more likely to recur (Xu et al., 2006). They start as vesicles, progressively transforming into vesicopustules, erosions, and ulcers (Corey, 1983).

**TABLE 2:** Comparison of neurological symptoms among Monkeypox, Smallpox, Varicella, and HSV

Symptoms	Monkeypox	Smallpox	Varicella	HSV‐1	HSV‐2
Fever	+	+	+	+	+
Headache	+	+		+	+
Blindness	+	+		+	+
Encephalitis	+	+	+	+	+
Seizures	+	+	+	+	+
Focal neurological deficits		+	+	+	
Delirium		+			
Hallucinations		+			
Acute cerebellar ataxia			+		
Myelitis			+	+	+
Facial paralysis			+^a^	+^b^	
Meningitis			+	+	+
Postherpetic neuralgia			+		
Autonomic dysfunction				+	
Radiculopathy					+

^a^ Ramsay Hunt Syndrome.

^b^ Bell's Palsy (Murakami, 1996).

HSV can enter the brain through the olfactory and trigeminal nerves to cause neurological complications (Steiner & Benninger, 2018). Encephalitis, typically involving the temporal lobe, is one of the dreaded complications of HSV‐1 (Whitley, 2006). Aseptic meningitis is more common with HSV‐2 (Kupila et al., 2006). Some unique psychiatric manifestations help differentiate it from other possible diagnoses. Hypomania may be observed initially due to inflammation of the limbic system or inferomedial temporal lobe (Fisher, 1996). Klüver–Bucy syndrome is a rare manifestation of HSV encephalitis characterized by hypersexuality and a loss of normal anger and fear responses (Hart et al., 1986). Autonomic dysfunction, although rare, is another differentiating feature of HSV‐1 infection (Steiner & Benninger, 2018). Neuropathy of the seventh cranial nerve is a common feature of HSV and VZV, presenting as Ramsay Hunt Syndrome and Bell's Palsy, respectively (Bozzola et al., 2012; Murakami, 1996).

Encephalitis is a common feature among all the differentials. Unique neurological manifestations of MPX are yet to be described. Hence, comparing the characteristic skin lesions and the community prevalence of viral diseases might be of greater help in narrowing the differentials.

## 6 NEUROLOGICAL COMPLICATIONS OF MPX VACCINES

Neurological complications are known to be associated with some vaccines including smallpox, mumps, measles, and rubella vaccine. With various poxviral vaccines repurposed to be used for MPX prevention, the risk of vaccine‐induced neurological complications cannot be ignored. Neurological complications have been widely reported with the smallpox vaccine (Guarner et al., 2022). ACAM2000 vaccine is approved for smallpox immunization and has been approved for use against MPX under an Expanded Access Investigational New Drug (EA‐IND) protocol (Abrahams & Kaufman, 2004). JYNNEOS, on the other hand, is the only vaccine on the market that has been approved by the FDA to protect against MPX (Guarner et al., 2022). Neurological complications have been reported for both vaccines, especially ACAM2000. Patients with immunosuppression, human immunodeficiency virus infection, eczema, or pregnancy should not receive preexposure vaccination. Individuals who are more liable for complications should be identified and precautions should be taken to keep them from coming into contact with those who have active vaccination lesions (Cleri et al., 2003).

Vaccinia strains can differ in their neurovirulence, for instance, when comparing Dryvax^®^ to ACAM1000, six young adult monkeys were inoculated with Dryvax^®^ or ACAM1000, and 50% of the monkeys who received Dryvax^®^ developed neurological complications, while non among the monkeys that received ACAM1000 develop any neurological complications. The neurological illness observed in the Dryvax^®^ group included severe meningitis (Monath et al., 2004). While ACAM2000 has been linked to new‐onset seizures, the third‐generation smallpox vaccination, MVA, is considered to be a more attenuated vaccine and a safer option than previous generations (WHO, 2018).

The most dreaded neurological complication of these vaccines is ADEM (Huynh et al., 2008; Sejvar, 2005). Vaccination with smallpox vaccines has been linked to several neurological problems, the most concerning being PVEM. Postvaccinal encephalopathy is more prevalent in children under the age of two (Booss & Davis, 2003). After 1–2 days, initial symptoms include fever, dizziness, and tiredness. Some people have high fevers together with neurological symptoms such as limb paralysis, urine retention, and seizures (Booss & Davis, 2003). PVEM mostly occurs after vaccination for the first time rather than after revaccination, while the incidence varies from 2 to 1219 cases following primary vaccination per million. PVEM can occur at all ages, although most primary vaccination against smallpox occurs at an age below 2 years; therefore, the incidence of PVEM is higher among this age group (Booss & Davis, 2003; Henderson & Moss, 1999). According to a study conducted by James J. Sejvar et al., the incidence of neurological adverse events following smallpox vaccination was 214 cases, with headache as the most frequent symptom followed by nonserious limb paresthesia, meningitis, encephalitis, Bell's palsy, seizures, and Guillain–Barré syndrome (Sejvar, 2005).

Neurological manifestations of LC16 vaccines were assessed in a research project in which rabbits were administered the attenuated and unattenuated Lister strains; researchers found that rabbits injected with unattenuated Lister strain suffered from encephalitis, while no cases of encephalitis were observed among attenuated Lister strains (Kenner et al., 2006).

However, current vaccination experience suggests that it has a very low risk of neurologic complications and does not cause exacerbations of chronic neurologic illnesses (Abrahams & Kaufman, 2004; Guarner et al., 2022). The overall prevalence of these vaccine‐induced neurological complications is very low and the benefit of these vaccines largely outweighs the associated risk, but an understanding of these possible complications is integral to patient management, especially in the scenario of a widespread outbreak.

## 7 EVOLVING MANAGEMENT STRATEGIES

The impact of neuropsychiatric manifestations of any viral disease cannot be understated. Cases from around the world have reported such manifestations for MPX, and have highlighted the need for developing guidelines and strategies for their management. The symptoms and presentations have shown significant variation on a case‐to‐case basis and thus multiple treatment strategies have been employed (Table 1). The approach to treatment has mostly been individualized as per the symptoms and severity of the disease. Most cases have just required medications for pain relief and antiviral therapy, while some serious ones landing into encephalitis have required intubation and ventilatory support. Oral antiviral medications for MPX include Cidofovir and Tecovirimat (Farahat et al., 2022). Corticosteroids may be considered for an ADEM‐like presentation, keeping in mind the possible immunosuppressive complications (Pastula et al., 2022). Vaccinia immune globulin can promptly be employed for treatment as well. Both MPX and smallpox are preventable by vaccines like the JYNNEOS vaccine and ACAM2000 (for those above 18 years of age) (Chopra et al., 2022; Shafaati & Zandi, 2022). Smallpox vaccines have been seen to provide about 85% or greater protection against MPX (Abrahams & Kaufman, 2004).

The psychiatric complications seem to be a consequence of events surrounding the disease process, including isolation, stigmatization, physical pain, and disfigurement. The healthcare providers must consider providing adequate pain management, psychological counseling, preserving human connections through virtual means, and prevention and management of dermal scarring as additions to the management plan. In addition, the dissemination and amplification of accurate public health information are vital in dispelling fear and misconceptions related to MPX. A troubling development during the recent COVID‐19 pandemic was a rise in Sinophobia (Cheah et al., 2020). The language and headlines used in news outlets and social media were major contributors to psychological disturbances and were shown to increase the trauma, rather than help to overcome it (Sethi et al., 2022). The MPX outbreak threatens a similar rise in homophobia, stigmatizing an already vulnerable community, through targeted headlines and media language (Bragazzi et al., 2022). It should be emphasized that direct contact with infectious scabs, body fluids, and sores is the main mode of transmission of the virus (CDC, 2022b). Sensitivity should be exercised by health organizations and news outlets while reporting on demographic data.

## 8 CONCLUSION

The neuropsychiatric manifestations of MPX are probably underreported and warrant due attention. With better reporting, the literature will soon evolve on long‐term complications as well. The government and private health agencies must promote better reporting of such complications and the use of management strategies. Integrated and planned guidelines can help early diagnosis and evidence‐based response. Not only the healthcare providers but also the patients and general population need to be educated and informed of possible complications. Patient education and screening programs can thus play a game‐changing role. Mental health programs must be constituted to help prevent the impacts of psychiatric manifestations. We must remember that we cannot imagine reaching close to the true definition of “health,” unless we promote and establish measures of comprehensive health care.

## 9 FUTURE PERSPECTIVES AND RECOMMENDATIONS

Medical professionals must be familiar with the neurological complications of MPX and the vaccines allowing early diagnosis and treatment. Future studies are warranted to explore the neurological complications of MPX and the vaccines and contribute more data to understand their prevalence, impact, and prognosis. Policymakers should raise awareness of the problem and encourage stakeholders to collaborate on a solution.

## AUTHOR CONTRIBUTIONS

Conceptualization and investigation: Yashendra Sethi, Pratik Agarwal, Summaiya Waheed, and Sajeda Ghassan Matar. Resources: Yashendra Sethi and Hamsa Murli. Data curation: Yashendra Sethi. Writing—original draft preparation: Yashendra Sethi, Pratik Agarwal, Hamsa Murli, Summaiya Waheed, Sajeda Ghassan Matar, and Mohamed Baklola. Writing—review and editing: Yashendra Sethi, Pratik Agarwal, Hamsa Murli, Summaiya Waheed, Sajeda Ghassan Matar, Mohamed Baklola, Hitesh Chopra, Talha Bin Emran, and Elfatih A. Hasabo. Visualization: Yashendra Sethi, Pratik Agarwal, Hamsa Murli, Summaiya Waheed, Sajeda Ghassan Matar, Mohamed Baklola, and Talha Bin Emran. Supervision: Yashendra Sethi. Project administration: Yashendra Sethi and Talha Bin Emran. Drafting the manuscript: Elfatih A. Hasabo. All authors have read and agreed to the published version of the manuscript.

## Funding information

This research received no external funding.

## CONFLICT OF INTEREST STATEMENT

The authors declare no conflicts of interest.

### PEER REVIEW

The peer review history for this article is available at https://publons.com/publon/10.1002/brb3.2934.

## Data Availability

Not applicable.
